# Sodium–Glucose Cotransporter 2 Inhibitors in Underweight Patients with Heart Failure: A Case Series

**DOI:** 10.3390/jcm15052027

**Published:** 2026-03-06

**Authors:** Masaki Nakagaito, Teruhiko Imamura, Toshihide Izumida, Makiko Nakamura, Koichiro Kinugawa

**Affiliations:** Second Department of Internal Medicine, University of Toyama, Toyama 930-0194, Japan; mgaito128@gmail.com (M.N.); itoshi700@gmail.com (T.I.); nakamuramk1979@gmail.com (M.N.); kinugawa0422@gmail.com (K.K.)

**Keywords:** heart failure, SGLT2 inhibitor, underweight, body mass index, adverse event

## Abstract

**Background:** Sodium–glucose cotransporter 2 inhibitors (SGLT2i) reduce mortality and morbidity in patients with heart failure (HF). However, their efficacy and safety in underweight patients remain uncertain. This study aimed to evaluate the efficacy and safety of SGLT2i in underweight patients with HF. **Methods:** This study was a single-center, prospective observational study designed to assess the efficacy of SGLT2i therapy in underweight patients with HF. The primary outcome was a composite of unplanned hospitalization for HF or death from cardiovascular causes. A key secondary outcome was hospitalization from any cause. **Results:** This study enrolled 131 consecutive patients with a body mass index (BMI) > 18.5 kg/m^2^ hospitalized for HF between December 2020 and October 2023. The median age of the study population was 81 (73–87) years, and 60% were female. Baseline BMI was 17.2 (16.0–17.9) kg/m^2^. Of these, 28 patients initiated SGLT2i during index hospitalization, while the remaining 103 did not receive SGLT2i. Over a median of 20.4 months of follow-up, the primary outcome occurred in 6 of 28 patients (21.4%) with SGLT2i and 22 of 103 patients (21.4%) without SGLT2i (*p* = 0.758). All-cause hospitalizations occurred in 23 of 28 patients (82.1%) with SGLT2i and 65 of 103 patients (63.1%) without SGLT2i (*p* = 0.009). Patients receiving SGLT2i showed a significant decrease in BMI at discharge, 1 month after discharge, and 3 months after discharge compared with those without SGLT2i (*p* < 0.05 for each time point). **Conclusions:** SGLT2i in underweight patients with HF may not reduce cardiovascular event risk and may be associated with a higher rate of overall hospitalizations.

## 1. Introduction

Heart failure (HF) has become a global pandemic, with its prevalence steadily increasing due to aging populations, improved survival from myocardial infarction, and the rising burden of lifestyle-related diseases [1]. HF is now one of the leading causes of hospitalization and mortality worldwide, particularly among the elderly [2]. Sodium–glucose cotransporter 2 inhibitor (SGLT2i) was initially developed as an antihyperglycemic agent for the management of type 2 diabetes mellitus (DM). However, large-scale randomized controlled trials (RCTs) have demonstrated that SGLT2i significantly reduced the risk of cardiovascular events, particularly HF hospitalization, irrespective of diabetes status or left ventricular ejection fraction (LVEF) [3,4,5,6]. Accordingly, dapagliflozin and empagliflozin, one of SGLT2is, have been assigned a class I recommendation for patients with HF with reduced left ventricular ejection fraction (HFrEF) in European and US guidelines, and a class I recommendation has also been granted for patients with HF with preserved left ventricular ejection fraction (HFpEF) in European guidelines [7,8,9].

SGLT2i has demonstrated cardiovascular benefits via pleiotropic mechanisms, among which weight reduction has been proposed as a potential contributor [10,11]. While SGLT2i has demonstrated clinical benefits across a wide range of HF populations, the safety and efficacy in underweight patients remain less established. Given the potential for volume depletion, muscle wasting, caloric loss, and catabolic effects associated with SGLT2i, concerns have been raised regarding their safety profile in underweight patients with HF. In real-world clinical practice, low body weight is not uncommon among patients with HF, particularly in elderly individuals or those with advanced disease stages. This population often has more comorbidities such as frailty, malnutrition, and sarcopenia. Several previous studies suggest that underweight individuals may have a higher risk of mortality compared to those who are normal or overweight [12,13]. In real-world clinical practice, initiating SGLT2i in such underweight patients might theoretically be a particularly challenging decision. Conversely, the favorable metabolic and hemodynamic effects of SGLT2i, including diuresis, improvement of cardiac energy metabolism, and renal protection, may offer substantial therapeutic advantages even in this vulnerable group. Several sub-analyses have reported that the benefits of SGLT2i are consistently present across all body mass index (BMI) categories in patients with HF [14,15,16,17].

Most major clinical trials evaluating SGLT2i have underrepresented extremely underweight patients (those with a BMI < 18.5 kg/m^2^). Such patients can often be encountered, particularly in Asian countries, including Japan. Nevertheless, limited evidence is available regarding the efficacy and safety of SGLT2i in HF patients with extremely low body weight. Therefore, we postulated that the efficacy of SGLT2i in reducing cardiovascular events might be attenuated in patients with HF and marked underweight. This study aims to evaluate the clinical impact of SGLT2i therapy in this specific population, focusing on both beneficial outcomes and potential adverse effects.

## 2. Materials and Methods

This single-center, prospective observational study was designed to evaluate the efficacy of SGLT2i therapy initiated during the index hospitalization for HF in underweight patients. In the present analysis, hospitalization events associated with SGLT2i use were retrospectively examined. The study was conducted in compliance with the Declaration of Helsinki, and written informed consent was obtained from all participants. The protocol was approved by the Institutional Ethics Board of Toyama University Hospital (Toyama, Toyama, Japan).

### 2.1. Study Population

Consecutive underweight patients who were hospitalized for HF at a large academic center between December 2020 and October 2023 were involved in this study. Underweight was defined as a BMI less than 18.5 kg/m^2^ at the time of index discharge. The majority of the patients presented with New York Heart Association class III/IV symptoms upon admission. Participants were treated with guideline-directed medical therapy for HF, including beta-blockers, renin–angiotensin system inhibitors or angiotensin receptor-neprilysin inhibitors, mineralocorticoid receptor antagonists, and diuretics, if applicable. This study included the use of only two SGLT2is: dapagliflozin and empagliflozin.

Patients were excluded if they met any of the following criteria: age < 18 years, hemodialysis or end-stage renal failure with estimated glomerular filtration rate < 15 mL/min/1.73 m^2^, implantation of a durable left ventricular assist device or prior heart transplantation, pregnancy or breastfeeding. Patients who were already taking any type of SGLT2i at the time of index admission were also excluded. Adjustments to medical therapy were permitted according to real-world clinical practice.

### 2.2. Study Design

To evaluate the clinical impact of SGLT2i in underweight patients with HF, we compared the hospitalization events between the participants who received SGLT2i during index hospitalization and those who did not receive the drug. Additionally, we compared the changes in BMI and plasma B-type natriuretic peptide (BNP) levels during index hospitalization between both patient groups to investigate the effects of SGLT2i in the acute phase of HF. Moreover, to examine the sustained effects of SGLT2i on body weight reduction and HF progression, we additionally examined the trajectory of BMI and BNP at 1 month and 3 months after index discharge.

Because the study population consisted of patients hospitalized for decompensated HF, day 0 was defined as the date of index discharge in order to reduce selection bias. Patients were censored at the time of death or loss to follow-up. Patients who were receiving SGLT2i were censored at the time of discontinuation, and those who were not receiving SGLT2i were censored at the time of initiation after index discharge. Data for patients who did not have a primary end-point event were censored on the last day they were known to have been free of the event. All patients were followed for up to 24 months or until the end of the observation period.

The primary outcome was a composite of unplanned hospitalization for HF or death from cardiovascular causes. A key secondary outcome was a hospitalization from any cause. The hospitalization events from any cause included those for non-cardiovascular causes. The additional secondary outcomes were a death from any cause and a hospitalization for HF. In addition, we assessed the change in BMI and BNP levels during index hospitalization, as well as the changes between admission and 1 month and 3 months after index discharge.

### 2.3. Data Collection

Baseline characteristics were retrieved at index discharge, including demographic, laboratory, and medication data. The BMI was calculated by dividing the total body weight (kilograms) by height squared (meters). Data on BMI and BNP were retrieved at the time of both hospital admission and discharge. Glomerular filtration rate was calculated using the guidelines from the Chronic Kidney Disease Epidemiology Collaboration. The calculation formula of the geriatric nutritional risk index (GNRI) was as follows: GNRI = 14.89 × serum albumin (g/dL) + 41.7 × BMI/22. Standard echocardiographic findings during index hospitalization were retrieved. We defined HFrEF as LVEF < 40%. DM was defined as a glycated hemoglobin level ≥ 6.5% or the use of antidiabetic medications. Loop diuretic doses were expressed as furosemide equivalents.

Follow-up information on the clinical course was obtained from our hospital’s medical records, including data on rehospitalization and mortality after discharge. In order to assess the safety of SGLT2i in underweight patients with HF, we examined the reasons for discontinuation among those in whom the drug was withdrawn. For events not recorded in our institutional database, information on hospitalizations and deaths was obtained from referral documents from other hospitals. Patients whose clinical outcomes could not be confirmed using these methods were excluded from the analysis.

### 2.4. Statistical Analyses

All statistical computations were executed employing JMP^®^ 18 (SAS Institute Inc., Cary, NC, USA). A two-sided *p*-value < 0.05 was considered statistically significant. Continuous variables are presented as medians with interquartile ranges (IQRs) and were compared using the Wilcoxon rank-sum test. Categorical variables are expressed as counts and percentages and were compared using the chi-square test.

The cumulative incidence of clinical events over 2 years was estimated using the Kaplan–Meier method, and intergroup differences were evaluated with the log-rank test. Associations between clinical variables and outcomes were assessed using univariable and multivariable Cox proportional hazards models, with hazard ratios (HRs) and 95% confidence intervals (CIs) reported. Variables entered into the univariable analyses included baseline patient characteristics, HF etiology, laboratory parameters that differed significantly between groups, and medications at discharge, including SGLT2 inhibitors. Variables with *p* < 0.05 in the univariable analyses were subsequently included in the multivariable models.

## 3. Results

### 3.1. Follow-Up and Patient Characteristics

**Figure 1:**
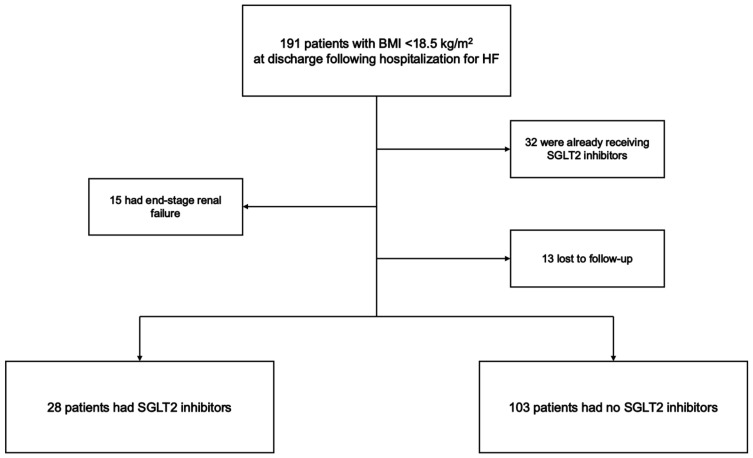
Patient flow chart.

191 consecutive underweight patients were hospitalized for HF. Of them, 32 were already taking SGLT2i upon admission, 15 had end-stage renal failure, and 13 patients were lost to follow-up. Finally, a total of 131 patients were included in this study (Figure 1).

Table 1 lists the baseline characteristics. The median age was 81 (73–87) years and 60% were women. Baseline BMI was 17.2 (16.0–17.9) kg/m^2^. HFrEF (LVEF < 40%) was present in 31 patients (24%). DM was observed in 19 patients (15%), all of whom had type 2 DM. 66 patients (50%) had a baseline clinical frailty scale (CFS) score of 4 to 9. The median baseline BNP level was 178 (93–407) pg/mL. All diuretics were administered orally.

Of these, SGLT2i were initiated in 28 patients, whereas it was not initiated in 103 patients during the index hospitalization. Baseline characteristics between the two groups were compared in Table 1. Patients with SGLT2i were younger and had a higher prevalence of ischemic heart disease, DM, and HFrEF. In patients with SGLT2i, who had a higher prevalence of HFrEF, the prescription rates of renin–angiotensin system inhibitors/angiotensin receptor-neprilysin inhibitors or mineralocorticoid receptor antagonists were higher. The CFS did not differ between the groups; however, patients with SGLT2i had a significantly higher GNRI. No significant differences in both BMI and plasma BNP were observed between the two groups.

### 3.2. Clinical Outcomes

The median hospitalization duration was 14 (8–27) days, and there was no statistically significant difference in the in-hospital duration between the two groups. Following index discharge, patients were followed for a median period of 612 (422–730) days. While the median follow-up period did not differ significantly between the two groups, the duration of follow-up varied across individual participants.

**Figure 2:**
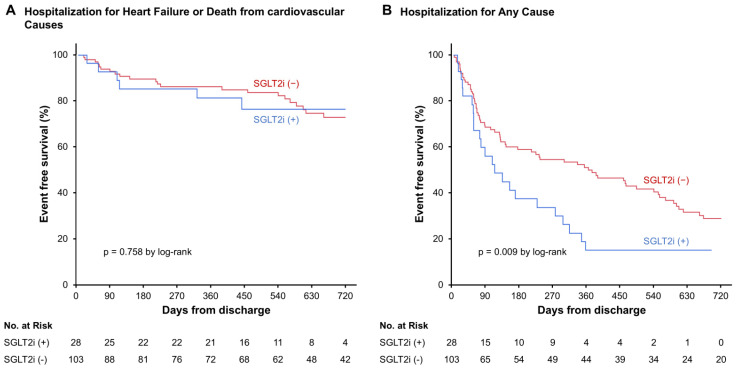
Kaplan–Meier curves for (**A**) a composite of unplanned hospitalization for heart failure or death from cardiovascular causes, (**B**) hospitalization for any cause.

**Figure 3:**
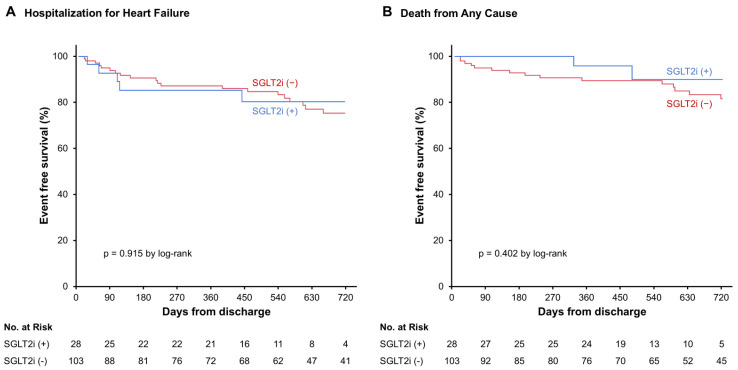
Kaplan–Meier curves for (**A**) hospitalization for heart failure, (**B**) death from any cause.

The cumulative incidence of rehospitalization for HF or death from cardiovascular causes was not significantly different (Figure 2A), but the cumulative incidence rate of hospitalization for any cause was significantly higher in patients who received SGLT2i than those without SGLT2i (*p* = 0.009; Figure 2B). The multivariable analysis demonstrated that the SGLT2i use was independently associated with the hospitalization for any cause (hazard ratio 1.90, 95% confidence interval 1.16–3.13), together with the lower prescription rate of beta-blockers (*p* < 0.050, respectively; Table 2). The cumulative incidences of rehospitalization for HF and those for all-cause death were not significantly different between the two groups, respectively (*p* > 0.05 for both; Figure 3A,B).

### 3.3. Causes of First Rehospitalizations

The events leading to all-cause rehospitalization are presented in Table 3. The most frequent cause of rehospitalization was cardiac catheterization, while HF accounted for approximately 20% of all hospitalizations.

In the SGLT2i-treated group, no hospitalizations related to dehydration or urinary tract infection attributable to SGLT2i were observed. Conversely, hospitalizations due to frailty occurred more frequently in patients who received SGLT2i than in those without SGLT2i, which warrants careful consideration (7% [2/28] versus 1% [1/103], *p* = 0.053).

### 3.4. Trajectory of BMI and BNP

At 1 month after discharge, BMI was measured in 16 patients with SGLT2i and 57 patients without SGLT2i, while at 3 months after discharge, BMI was measured in 24 patients with SGLT2i and 66 patients without SGLT2i. Similarly, plasma BNP was measured in 17 patients with SGLT2i and 55 patients without SGLT2i at 1 month after discharge, and in 23 patients with SGLT2i and 66 patients without SGLT2i at 3 months after discharge, by excluding those who were censored or those with data deficiency.

**Figure 4:**
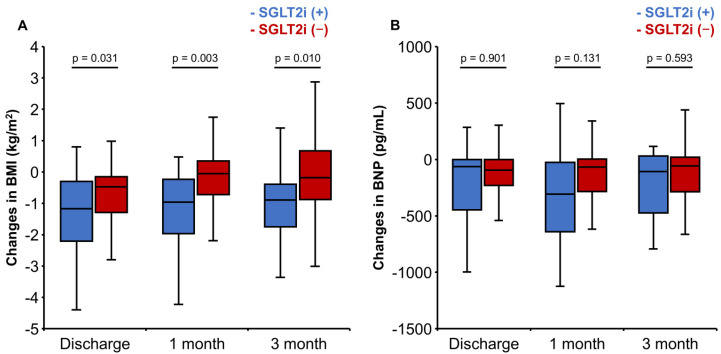
Changes in (**A**) body mass index and (**B**) B-type natriuretic peptide from admission to discharge, 1 month after discharge, and 3 months after discharge.

Patients treated with SGLT2i showed a significantly greater reduction in BMI compared to those who were not treated with them (−1.17 [−2.21–−0.30] versus −0.47 [−1.28–−0.15], *p* = 0.031; Figure 4A). Furthermore, the differences in BMI changes were similarly observed at both 1 month and 3 months after discharge. Conversely, changes in BNP levels showed no significant differences between the two groups during hospitalization or after discharge (Figure 4B).

### 3.5. Feasibility

During the observation period, five (17.9%) of the 28 patients receiving SGLT2i discontinued the medication. Four of them discontinued due to drug-related adverse events, and one discontinued the medication based on personal judgment. The drug-related adverse events that led to the discontinuation of SGLT2i were dehydration in two patients, weight loss in one patient, and hypoglycemia in one patient. Meanwhile, among the 103 patients who were not initially treated with SGLT2i, 11 patients (10.7%) started SGLT2i after index discharge. These patients were censored at the time of discontinuation or initiation of SGLT2i, respectively.

## 4. Discussion

### 4.1. Study Findings

In this study, we investigated the impact of SGLT2i therapy in underweight patients with HF. While SGLT2i did not reduce the risk of hospitalization for HF or cardiovascular death, their use was associated with an increased risk of all-cause hospitalization. Furthermore, SGLT2i significantly reduced BMI during the index hospitalization for HF and throughout the 3-month period following index discharge, even in patients who were initially underweight.

### 4.2. Impact of SGLT2i in Underweight Patients

In subgroup and meta-analyses from RCTs, the cardiovascular benefits of SGLT2i have been consistently demonstrated regardless of body weight [14,15,16,17,18]. However, most of these analyses did not categorize underweight patients, and the effects of SGLT2i in this specific population remain unclear. This is likely due to the extremely small proportion of underweight patients enrolled in these RCTs, which accounted for only about 1% of the study populations. In DAPA-HF, only 87 patients (1.8%) were classified as extremely underweight, a considerably smaller number than in our study population. To date, no studies have specifically evaluated the efficacy and safety of SGLT2i on HF in underweight patients with a BMI < 18.5 kg/m^2^. To the best of our knowledge, this is the first study to investigate the clinical effects of SGLT2i in underweight patients with HF.

### 4.3. Impact of SGLT2i on the Primary Outcome

In the present study, the primary outcome of a composite of unplanned hospitalization for HF or death from cardiovascular causes did not differ between patients with and without SGLT2i treatment. In the post-hoc analysis of the EMPEROR-Reduced trial, the clinical benefits of empagliflozin versus placebo were consistently present across different BMI categories in patients with HFrEF [16]. However, in patients with a BMI less than 20 kg/m^2^, the incidence of cardiovascular events did not differ significantly between the empagliflozin and placebo groups in this sub-analysis. These findings are consistent with the results of the present study, suggesting that the cardiovascular benefits of SGLT2i may be attenuated in underweight patients. In contrast, a previous study reported that SGLT2i reduced all-cause mortality and cardiovascular death in HF patients with a BMI < 20 kg/m^2^ [19]. However, unlike our study, that study included patients with a prior history of SGLT2i use before enrollment, which may have contributed to baseline imbalances in the BNP levels and renal function between the treatment groups. By excluding patients with a prior history of SGLT2i use, we ensured a more accurate assessment of SGLT2i efficacy. In this study, SGLT2i use was identified as an independent risk factor for all-cause hospitalization, irrespective of intergroup differences in patient characteristics, including age, nutritional status, and HF phenotype.

### 4.4. Diuretic Impact of SGLT2i

The primary action of SGLT2i is to promote urinary glucose excretion through inhibition of glucose reabsorption in the proximal renal tubules [20]. The weight loss associated with SGLT2i is presumed to result from the urinary glucose excretion, which induces a combination of natriuresis and osmotic diuresis, reduction in adipose tissue, and a potential decrease in skeletal muscle mass. In particular, the early phase of weight loss following SGLT2i initiation is mainly attributed to fluid volume reduction due to diuretic effects. In the present study, patients who received SGLT2i showed a significantly greater decrease in BMI compared to the patients who did not receive SGLT2i, which is likely associated with the diuretic effect of SGLT2i. In such cases, the weight loss observed with SGLT2i treatment may reflect a beneficial response contributing to hemodynamic compensation in patients with HF. Several clinical trials have reported the efficacy of SGLT2i even in hospitalized or recently discharged patients with HF [21,22,23]. The present findings suggest that SGLT2i exert beneficial acute-phase effects even in patients with a low BMI, underscoring the clinical significance of these agents in the management of HF regardless of body weight. However, the change in BNP did not differ significantly between patients with and without SGLT2i. BMI reduction during SGLT2i therapy may not simply be explained by the improvement of congestion.

### 4.5. Multimodal Impacts of SGLT2i

In this study, the weight-reducing effect of SGLT2i was prolonged and rather gradually enhanced for 3 months, probably reflecting not only the diuretic effect of SGLT2i alone but also its impact on energy deficit. The energy loss associated with glycosuria and the compensatory increase in gluconeogenesis may lead to amino acid depletion, enhanced skeletal muscle catabolism, potentially promoting sarcopenia, particularly in vulnerable populations [24]. Several previous studies reported some direct myocardial effects of SGLT2i, including improvement of the transduction of oxygen consumption into work efficiency under conditions of hyperketonemia, which is considered one of the cardioprotective mechanisms of this drug class [25].

Nevertheless, in patients with inherently low body fat, the beneficial effects of SGLT2i may be limited, and there are concerns about potential risks such as progression of frailty and the development of ketoacidosis. In this study, SGLT2i use was associated with a higher incidence of all-cause hospitalization. One possible explanation for this finding may be concerns specific to underweight patients, in whom SGLT2i could potentially exacerbate adverse effects such as sarcopenia, volume depletion, or malnutrition-related complications, leading to rehospitalization. Consistently, hospitalizations related to frailty occurred more frequently in patients who received SGLT2i than in those without SGLT2i. This finding suggests that, when SGLT2i are used in underweight patients, closer monitoring of frailty-related clinical parameters, such as body weight, nutritional status, and physical function, may be warranted. Conversely, no hospitalizations directly attributable to typical adverse effects of SGLT2i, such as dehydration, volume depletion, or urinary tract infection, were observed in the SGLT2i-treated group. Furthermore, in underweight patients, the excessive effects of SGLT2i may have contributed to hospitalizations that were not directly related to frailty, including those for HF. This mechanism may not only have increased the all-cause hospitalization but also potentially attenuated the cardioprotective advantages of SGLT2i.

Importantly, because our cohort consisted of severely underweight patients with advanced HF, the rates of both cardiovascular and non-cardiovascular readmissions were high across the entire population, irrespective of SGLT2i use. These patients have inherently limited physiological reserve, making them susceptible not only to HF-related events but also to a wide range of non-cardiac complications. Further investigation is needed to determine whether hospitalization due to cardiovascular and non-cardiovascular causes can be prevented in this patient population, and to identify effective strategies for achieving this goal.

### 4.6. Feasibility of SGLT2i

SGLT2i was terminated for reasons other than death in 5 out of the 29 patients in whom the medication was initiated in this study. Our withdrawal rate of 17.2% was comparable to the previously reported rates of 10.5–23.2% in RCTs [3,4,5,6]. On the other hand, a previous RCT showed that adverse events leading to discontinuation of therapy were highest in the BMI < 20 kg/m^2^ category [16]. Given that a higher incidence of adverse events has been observed in underweight patients irrespective of SGLT2i use, it is imperative to closely monitor the clinical course of such patients, particularly when SGLT2i is administered. Further studies are warranted to clarify the risk-benefit profile of SGLT2i in this specific subgroup, as current evidence is limited and largely derived from post hoc analyses or underpowered subgroups within RCTs.

### 4.7. Limitations

This study has several limitations. First, it was a single-center observational study with a relatively small overall sample size and a limited number of primary outcome events. Furthermore, because the study population consisted primarily of underweight patients, the initiation rate of SGLT2i tended to be low, at 21.4%. The small sample size and limited number of events resulted in insufficient statistical power to evaluate the primary outcome in this study. In addition, the decision to prescribe SGLT2i was made at the discretion of the attending physicians, which resulted in differences in baseline characteristics between patients with SGLT2i and those without SGLT2i. The comparisons between groups may have been influenced by selection bias, given the differences in baseline characteristics such as age and nutritional status. Notably, patients who received SGLT2i had a significantly higher proportion of HFrEF compared to those who did not receive the treatment. This is likely due to the publication of the EMPEROR-Preserved and DELIVER trials during the study period, which may have strongly influenced prescribing practices. Although several studies have reported no substantial difference in clinical outcomes among patients with HFrEF, HF with mildly reduced LVEF, and HFpEF, there have been no direct comparisons of the effects of SGLT2i between these phenotypes of HF [26]. Moreover, a detailed assessment of dementia, which has been reported to be associated with prognosis in frail patients, was not performed in this study. Given the relatively small number of patients treated with SGLT2i and the limited number of outcome events, performing robust propensity score matching would have substantially reduced the effective sample size and statistical power, potentially resulting in unstable estimates. Therefore, we refrained from conducting such analyses. The findings of the present study should be interpreted as exploratory and hypothesis-generating. Larger multicenter studies with adequate statistical power are warranted to confirm the findings of the present study.

## 5. Conclusions

Among underweight patients with HF, SGLT2i did not reduce the risk of HF readmission or cardiovascular death, and was rather associated with an increased incidence of all-cause hospitalization events. Although SGLT2i confers robust cardiovascular and renal benefits in broad populations, its use in underweight patients may necessitate more cautious assessment. The present findings suggest that, for underweight individuals, the balance between therapeutic benefits and the potential risk of frailty-related events might differ from that in the general HF population. This hypothesis warrants further investigation in prospective studies specifically designed to evaluate the interaction between baseline nutritional status, frailty severity, and the safety profile of SGLT2i.

## Figures and Tables

**Table 1:** Baseline characteristics.

	Total (*n* = 131)	SGLT2i (+) (*n* = 28)	SGLT2i (−) (*n* = 103)	*p* Value
Age, years	81 (73–87)	75 (72–81)	83 (75–87)	0.002 *
Male, *n* (%)	52 (40)	11 (39)	41 (40)	0.960
Body mass index, kg/m^2^	17.2 (16.0–17.9)	17.6 (17.0–18.0)	17.1 (15.8–17.9)	0.164
Systolic blood pressure, mmHg	102 (90–116)	99 (90–107)	103 (90–120)	0.260
Heart rate, beats per minutes	70 (63–77)	75 (63–80)	70 (63–77)	0.391
Diabetes mellitus, *n* (%)	19 (15)	8 (29)	11 (11)	0.017 *
Ischemic etiology, *n* (%)	25 (19)	10 (36)	15 (15)	0.012 *
Atrial fibrillation, *n* (%)	48 (37)	7 (25)	41 (40)	0.149
De novo heart failure, *n* (%)	46 (35)	13 (46)	33 (32)	0.157
New York Heart Association class III–IV, *n* (%)	62 (47)	14 (50)	48 (47)	0.750
Left ventricular ejection fraction, %	51 (40–64)	38 (32–53)	53 (43–65)	<0.001 *
Value of <40% (HFrEF), *n* (%)	31 (24)	15 (54)	16 (15)	<0.001 *
Clinical frailty scale 4–9, *n* (%)	66 (50)	15 (54)	51 (50)	0.703
GNRI	81.6 (75.3–86.7)	82.7 (78.7–89.7)	79.8 (73.9–85.8)	0.039 *
HbA1c, %	5.9 (5.6–6.3)	6.0 (5.6–6.5)	5.9 (5.6–6.2)	0.110
Hemoglobin, g/dL	10.9 (9.7–12.1)	11.4 (10.9–13.0)	10.5 (9.6–12.0)	0.002 *
Serum albumin, g/dL	3.3 (2.9–3.7)	3.4 (3.1–3.8)	3.2 (2.9–3.7)	0.090
Serum sodium, mEq/L	138 (135–140)	137 (135–140)	139 (136–140)	0.126
Serum potassium, mEq/L	4.3 (4.0–4.6)	4.4 (4.0–4.6)	4.3 (4.0–4.6)	0.904
eGFR, mL/minute/1.73 m^2^	52.4 (35.9–67.7)	54.3 (35.2–69.3)	51.9 (36.1–67.7)	0.743
Uric acid, mg/dL	5.1 (4.2–7.1)	4.5 (3.5–5.9)	5.3 (4.3–7.3)	0.018 *
Plasma BNP, pg/mL	178 (93–407)	189 (88–437)	172 (97–369)	0.522
Treatment				
Beta-blockers, *n* (%)	99 (76)	24 (86)	75 (73)	0.159
ACEI/ARB/ARNI, *n* (%)	114 (87)	28 (100)	86 (84)	0.021 *
Loop diuretics, *n* (%)	78 (60)	16 (57)	62 (60)	0.688
Dose of loop diuretics, mg/day	10 (0–20)	10 (0–20)	10 (0–20)	0.223
MRA, *n* (%)	75 (57)	22 (79)	53 (52)	0.010 *
Implantable cardioverter–defibrillator, *n* (%)	11 (8)	5 (18)	6 (6)	0.042 *
Cardiac resynchronization therapy, *n* (%)	10 (8)	5 (18)	5 (5)	0.022 *

eGFR, estimated glomerular filtration rate; HFrEF, heart failure with reduced ejection fraction (ejection fraction < 40%); GNRI, geriatric nutritional risk index; HbA1c, glycated hemoglobin; BNP, b-type natriuretic peptide; ACEI, angiotensin converting enzyme inhibitors; ARB, angiotensin receptor blockers; ARNI, angiotensin receptor-neprilysin inhibitors; MRA, mineralocorticoid receptor antagonists. * *p* < 0.050.

**Table 2:** Variables associated with hospitalization for any cause.

	All Patients (*n* = 131)					
	Univariable Analysis			Multivariable Analysis		
Variables	Hazard Ratio	95% CI	*p* Value	Hazard Ratio	95% CI	*p* Value
Age, years	1.02	1.00, 1.04	0.074			
Ischemic etiology, yes	1.05	0.61, 1.80	0.876			
Atrial fibrillation, yes	1.36	0.89, 2.10	0.158			
HFrEF, yes	0.95	0.57, 1.59	0.856			
Clinical frailty scale 4–9, yes	1.29	0.85, 1.97	0.229			
GNRI	1.02	0.99, 1.05	0.181			
Diabetes mellitus, yes	1.35	0.75, 2.44	0.320			
Hemoglobin, g/dL	1.07	0.94, 1.20	0.309			
eGFR, mL/min/1.73 m^2^	0.99	0.98, 1.00	0.064			
Uric acid, mg/dL	1.10	0.98, 1.23	0.099			
ln BNP	0.98	0.69, 1.39	0.890			
Beta-blockers, yes	0.58	0.37, 0.92	0.021 *	0.53	0.33, 0.85	0.008 *
ACEI/ARB/ARNI, yes	0.66	0.37, 1.17	0.159			
Loop diuretics, yes	1.09	0.71, 1.67	0.688			
MRA, yes	1.06	0.70, 1.62	0.782			
SGLT2i, yes	1.89	1.16, 3.07	0.010 *	2.07	1.27, 3.39	0.004 *

HFrEF, heart failure with reduced ejection fraction (ejection fraction < 40%); GNRI, geriatric nutritional risk index; eGFR, estimated glomerular filtration rate; BNP, b-type natriuretic peptide; ACEI, angiotensin converting enzyme inhibitors; ARB, angiotensin receptor blockers; ARNI, angiotensin receptor-neprilysin inhibitors; MRA, mineralocorticoid receptor antagonists. * *p* < 0.050.

**Table 3:** First hospitalization events after index discharge.

	Total Number (%) of Events		
	Total (*n* = 88)	SGLT2 (+) (*n* = 23)	SGLT2 (−) (*n* = 65)
Cardiac catheterization	26 (30)	8 (35)	18 (28)
Heart failure	19 (22)	4 (17)	15 (23)
Catheter ablation for cardiac arrhythmia	10 (11)	3 (13)	7 (11)
Pneumonia	4 (5)		4 (6)
Stroke	4 (5)	1 (4)	3 (5)
Frailty	3 (3)	2 (9)	1 (2)
Dehydration	2 (2)		2 (3)
Injury disease	2 (2)		2 (3)
Lung tumor	2 (2)		2 (3)
Peripheral arterial disease	2 (2)	1 (4)	1 (2)
Aortic disease	1 (1)		1 (2)
Autoimmune disease	1 (1)	1 (4)	
Gastric tumor	1 (1)	1 (4)	
Gastrointestinal bleeding	1 (1)		1 (2)
Hypoglycemia	1 (1)	1 (4)	
Hyponatremia	1 (1)		1 (2)
Implantation of cardiac implantable electronic device	1 (1)		1 (2)
Ischemic enteritis	1 (1)		1 (2)
Myelodysplastic syndrome	1 (1)		1 (2)
Ophthalmic surgery	1 (1)		1 (2)
Pancreatic cancer	1 (1)		1 (2)
Urinary tract infection	1 (1)		1 (2)
Valvular surgery	1 (1)		1 (2)
Sarcoidosis	1 (1)	1 (4)	

## Data Availability

The data presented in this study are available on request from the corresponding author. The data are not publicly available due to privacy.
